# Supplementary material for: Bulk-surface coupling identifies the mechanistic connection between Min-protein patterns in vivo and in vitro
Source: Nat Commun. 2021 Jun 3;12:3312. doi: 10.1038/s41467-021-23412-5 (PMC8175580; doi:10.1038/s41467-021-23412-5)
Supplement: Supplementary file 1 — Supplementary Information [file 41467_2021_23412_MOESM1_ESM.pdf]

# Supplementary Information for

## Bulk-surface coupling identifies the mechanistic connection between Min-protein patterns *in vivo* and *in vitro*

Fridtjof Brauns[*], Grzegorz Pawlik[*], Jacob Halatek[*], Jacob Kerssemakers, Erwin Frey[†], and Cees Dekker[†]

[*]These authors contributed equally.
[*]Correspondence to: frey@lmu.de, c.dekker@tudelft.nl

### This PDF file includes:

Supplementary Methods 1 to 4
Supplementary Discussions 1 and 2
Supplementary Table 1
Supplementary Figures 1 to 12
Supplementary References

### Other supplementary materials for this manuscript include the following:

Supplementary Movies 1 to 20

## Supplementary Method 1. Mathematical model, domain geometry, and parameters

We use the skeleton Min model established in Refs. [1–3]. The microchamber geometry is represented by a cuboid bulk of dimensions $L \times L \times H$ with membrane (reactive boundaries) at the top and bottom surface and no-flux boundary conditions at the remaining surfaces (Supplementary Fig. 2). Because in the model, the dynamics on the 2d membranes is coupled to to the 3d bulk, we refer to this as 2+3D geometry. Simulations in the full 2+3D geometry are computationally very costly. A reduced two-dimensional geometry, comprising a rectangular bulk of width $L$ and height $H$ with membrane at the top and bottom boundary, represents a slice through the 3d system. This 1+2D geometry allows us to perform simulations of large systems over long timescales and also simplifies analysis and presentation of the results.

In the following, the model and linear stability analysis are presented in the the 1+2D geometry to keep notation compact. Generalization to the 2+3D geometry is straightforward. It is convenient to choose coordinates that respect the geometry's top-bottom symmetry with the vertical coordinate $z = 0$ at the center. The membranes are at $z = \pm h$, where we introduce the half-height $h := H/2$ as a shorthand.

Let us denote membrane concentrations of MinD and MinDE complexes by $\mathbf{m}_\pm = (m_\mathrm{d}^\pm, m_\mathrm{de}^\pm)$, where $\pm$ represent the top and bottom membrane, respectively. The cytosol concentrations of MinD and MinE are denoted by $\mathbf{c} = (c_\mathrm{D}, c_\mathrm{DD}, c_\mathrm{E})$, where $c_\mathrm{D} = c_\mathrm{DT} + c_\mathrm{DD}$ is MinD's total bulk concentration and $c_\mathrm{DD}$, $c_\mathrm{DT}$ are the bulk concentrations of MinD-ADP and MinD-ATP respectively. Using the pair of variables $(c_\mathrm{D}, c_\mathrm{DD})$ instead of $(c_\mathrm{DT}, c_\mathrm{DD})$ to account for MinD' bulk concentrations has the advantage that the respective bulk equations decouple from each other. The bulk dynamics read

$$\partial_t \mathbf{c}(x,z,t) = D_c(\partial_x^2 + \partial_z^2)\mathbf{c} - \boldsymbol{\beta}\mathbf{c}, \tag{1}$$

where $\partial_t$ denotes the time derivative and $\partial_x$, $\partial_z$ denote the spatial derivatives in lateral and vertical direction respectively. The linear reaction term $\boldsymbol{\beta}\mathbf{c} = \mathrm{diag}(0, \lambda, 0)\mathbf{c}$ captures nucleotide exchange of MinD. Reactions at the membranes on the top and bottom surfaces ($z = \pm h$) lead to bulk flows normal to the surface

$$\mp D_c \partial_z \mathbf{c}|_{z=\pm h} = \boldsymbol{f}(\mathbf{m}_\pm, \mathbf{c}|_{z=\pm h}), \tag{2}$$

where the attachment-detachment flow is given by

$$\boldsymbol{f}(\mathbf{m}, \mathbf{c}) = \begin{pmatrix} -(k_\mathrm{D} + k_\mathrm{dD} m_\mathrm{d})(c_\mathrm{D} - c_\mathrm{DD}) \\ k_\mathrm{de} m_\mathrm{de} \\ k_\mathrm{de} m_\mathrm{de} - k_\mathrm{dE} m_\mathrm{d} c_\mathrm{E} \end{pmatrix}. \tag{3}$$

The remaining boundaries are equipped with no-flux (alternatively periodic) boundary conditions

$$\partial_x \mathbf{c}|_{x=0,L} = 0. \tag{4}$$

The membrane dynamics are

$$\partial_t \mathbf{m}_\pm(x,t) = D_m \partial_x^2 \mathbf{m}_\pm + \mathbf{r}(\mathbf{m}_\pm, \mathbf{c}|_{z=0}), \tag{5}$$

with the reaction term

$$\mathbf{r}(\mathbf{m}, \mathbf{c}) = \begin{pmatrix} (k_\mathrm{D} + k_\mathrm{dD} m_\mathrm{d})(c_\mathrm{D} - c_\mathrm{DD}) - k_\mathrm{dE} m_\mathrm{d} c_\mathrm{E} \\ k_\mathrm{dE} m_\mathrm{d} c_\mathrm{E} - k_\mathrm{de} m_\mathrm{de} \end{pmatrix}, \tag{6}$$

and no-flux (alternatively periodic) boundaries $\partial_x \mathbf{m}_\pm|_{x=0,L} = 0$.

These dynamics conserve the total average densities of MinD and MinE

$$\bar{n}_\mathrm{D} = \frac{1}{2hL}\int_0^L \mathrm{d}x \left[ m_\mathrm{d}(x,t) + m_\mathrm{de}(x,t) + \int_{-h}^{+h} \mathrm{d}z\, c_\mathrm{D}(x,z,t) \right], \tag{7a}$$

$$\bar{n}_\mathrm{E} = \frac{1}{2hL}\int_0^L \mathrm{d}x \left[ m_\mathrm{de}(x,t) + \int_{-h}^{+h} \mathrm{d}z\, c_\mathrm{E}(x,z,t) \right]. \tag{7b}$$

**Supplementary Table 1. Default parameters of the Min skeleton model used throughout this study based on the parameters used in [3].**

| Symbol | Value | Unit | Description |
|---|---|---|---|
| $\bar{n}_\mathrm{D}$ | 400 | $\mu m^{-3}$ | Total MinD density (spatial average) |
| $\bar{n}_\mathrm{E}$ | varied | $\mu m^{-3}$ | Total MinE density (spatial average) |
| $H$ | varied | µm | Bulk height |
| $\lambda$ | 6 | $s^{-1}$ | Nucleotide exchange |
| $k_\mathrm{D}$ | 0.1 | $\mu m\, s^{-1}$ | Spontaneous MinD attachment |
| $k_\mathrm{dD}$ | 0.1 | $\mu m^3\, s^{-1}$ | MinD self-recruitment |
| $k_\mathrm{dE}$ | 0.15 | $\mu m^3\, s^{-1}$ | Recruitment of cytosolic MinE by membrane-bound MinD |
| $k_\mathrm{de}$ | 0.5 | $s^{-1}$ | MinDE complex dissociation |
| $D_m$ | 0.013 | $\mu m^2\, s^{-1}$ | Membrane diffusion |
| $D_c$ | 60 | $\mu m^2\, s^{-1}$ | Cytosolic diffusion |

**Parameters.** Our choice of parameters given in Supplementary Table 1 is based on the parameters used in [3]. Note that only the diffusion constants, nucleotide exchange rate $\lambda$, and total protein densities are known from experiments [4, 5]. We slightly adapt the kinetic rates from [3] such that the regime of pattern formation (indicated by linear instability of the homogeneous steady state) extends down to small bulk heights ($H \approx 1\,\mu m$) as was observed experimentally.

Note that the minimal model used here does not exhibit instabilities for E:D ratios larger than unity. For reference we note that an extended model, that also captures the MinE-switching, has a much larger range of instability extending to E:D ratios far above unity [6]. For the purpose of this study, the minimal model is sufficient however, because we are interested only in the qualitatively different mechanisms (oscillation modes) underlying pattern formation.

## Supplementary Method 2. Linear stability analysis

To identify the regimes in which the three different oscillation modes are active, we performed linear stability analysis. A mode is active if the homogeneous steady state is unstable against this mode. The general procedure for the linear stability analysis of a (homogeneous) steady state is as follows: First, one linearizes the dynamics in the vicinity of the steady state. The linearized dynamics can then be solved by analytically finding a set of spatial eigenmodes that grow/decay exponentially in time. This ansatz reduces the linear stability problem to a system of linear algebraic equations, and the growth rates $\sigma$ as a function of the spatial wavenumber $q$ are obtained as solutions of this system's characteristic equation. The relationship $\sigma(q)$ is called *dispersion relation* and encodes the stability properties of local ($q = 0$) and lateral ($q > 0$) modes. Here, we use the term local as antonym to lateral. The local modes are those that do not require lateral coupling. In the Min system the local modes are driven by vertical concentration gradients in the bulk. We therefore call them *vertical* modes, while local modes, and respectively local instabilities, is a more general term.

The equations for the homogeneous steady states and the characteristic equation, which yields the dispersion relation, can efficiently be solved numerically (using, for instance, *Mathematica* [7]).

In the following, we describe how this general procedure is carried out for the bulk-surface coupled reaction diffusion system Eqs. (1)–(5) in 1+2D slice geometry. The results generalize immediately to 2+3D geometry, since one can pick a direction of interest in the lateral dimensions without loss of generality. Along this direction, the governing equations are identical to the 1+2D geometry.

**Finding the laterally homogeneous steady states.** A steady state that is *laterally* homogeneous in the direction along the membrane (i.e. $\partial_x \widetilde{\mathbf{m}}_\pm = 0$ and $\partial_x \widetilde{\mathbf{c}}|_{z=\pm h} = 0$) may still have gradients normal to the membrane caused by cytosolic nucleotide exchange. To account for this fact, we refer to such states as *laterally homogeneous* steady states (LHSS).

In the bulk, the steady state condition reads

$$0 = D_c(\partial_x^2 + \partial_z^2)\widetilde{\mathbf{c}} - \boldsymbol{\beta}\widetilde{\mathbf{c}},$$

which can be solved by a separation of variables

$$\widetilde{c}_i(x,z) \propto \widetilde{X}_i(x)\widetilde{Z}_i(z),\ i = \{\mathrm{D}, \mathrm{DD}, \mathrm{E}\}.$$

Hence, homogeneity at the membrane implies $\widetilde{X}(x) = \text{const}$, and thus $\partial_x\widetilde{\mathbf{c}} = 0$ in the entire bulk.

For the components $c_\mathrm{D}$ and $c_\mathrm{E}$ the bulk dynamics is purely diffusive, so $\widetilde{Z}_{\mathrm{D,E}}(z) = C^\mathrm{s}_{\mathrm{D,E}} + C^\mathrm{as}_{\mathrm{D,E}}z$. For the $c_\mathrm{DD}$ component, we have

$$0 = D_c Z''_\mathrm{DD}(z) - \lambda Z_\mathrm{DD}(z), \tag{8}$$

which is solved by a superposition of exponentials $e^{\pm\sqrt{\lambda/D_c}\,z}$. Owing to the up-down symmetry of the system, we can express the solution as a sum of a symmetric and an antisymmetric contribution

$$Z_\mathrm{DD}(z) = C^\mathrm{s}_\mathrm{DD}\frac{\cosh(\sqrt{\lambda/D_c}\,z)}{\cosh(\sqrt{\lambda/D_c}\,h)} + C^\mathrm{as}_\mathrm{DD}\frac{\sinh(\sqrt{\lambda/D_c}\,z)}{\sinh(\sqrt{\lambda/D_c}\,h)}.$$

Both terms are normalized such that the MinD-ADP concentration at the top/bottom membrane is given by $C^\mathrm{s}_\mathrm{DD} \pm C^\mathrm{as}_\mathrm{DD}$. In the following, we consider only the vertically symmetric steady states, i.e. $C^\mathrm{as}_i = 0$ and $\widetilde{\mathbf{m}}_- = \widetilde{\mathbf{m}}_+ =: \widetilde{\mathbf{m}}_\mathrm{s}$.

Plugging the symmetric bulk profile into the bulk-surface coupling Eq. (2) and the membrane dynamics Eq. (5), we obtain the set of equations

$$\mathbf{r}(\widetilde{\mathbf{m}}_\mathrm{s}, \{C^\mathrm{s}_i\}) = 0, \tag{9a}$$
$$f_\mathrm{D}(\widetilde{\mathbf{m}}_\mathrm{s}, \{C^\mathrm{s}_i\}) = 0, \tag{9b}$$
$$f_\mathrm{E}(\widetilde{\mathbf{m}}_\mathrm{s}, \{C^\mathrm{s}_i\}) = 0, \tag{9c}$$
$$f_\mathrm{DD}(\widetilde{\mathbf{m}}_\mathrm{s}, \{C^\mathrm{s}_i\}) = D_c\sqrt{\lambda/D_c}\tanh\left(\sqrt{\lambda/D_c}\,h\right)C^\mathrm{s}_\mathrm{DD}. \tag{9d}$$

Together with the total density constraints (cf. Eq. (7))

$$\bar{n}_\mathrm{D} = C^\mathrm{s}_\mathrm{D} + \frac{1}{h}(\widetilde{m}^\mathrm{s}_\mathrm{d} + \widetilde{m}^\mathrm{s}_\mathrm{de}), \tag{10a}$$
$$\bar{n}_\mathrm{E} = C^\mathrm{s}_\mathrm{E} + \frac{1}{h}\widetilde{m}^\mathrm{s}_\mathrm{de}, \tag{10b}$$

these equations determine the LHSS. This set of algebraic equations can be solved numerically (e.g. in Mathematica [7], using the built-in function `NSolve[]`).

Asymmetric steady states can be determined analogously, where the bulk-surface coupling and membrane reactions need to be solved on both membranes individually, so there will be two equations for each of the equations (9a)–(9d) that determine the symmetric steady states.

**The linearized dynamics for small perturbations.** Linear stability of a steady state is studied by calculating the growth rate of small perturbations $(\delta\mathbf{m}_\pm, \delta\mathbf{c})$ around the steady state. (We restrict our analysis to the linear stability of vertically symmetric steady states here.[1]) For sufficiently small perturbations, the dynamics can be linearized

$$\partial_t\delta\mathbf{c}(x,z,t) = D_c(\partial_x^2 + \partial_z^2)\mathbf{c} - \boldsymbol{\beta}\mathbf{c}, \tag{11a}$$
$$-D_c\partial_z\delta\mathbf{c}|_{z\pm h} = \boldsymbol{f}_\mathbf{m}\delta\mathbf{m}_\pm + \boldsymbol{f}_\mathbf{c}\delta\mathbf{c}|_{z\pm h}, \tag{11b}$$
$$\partial_t\delta\mathbf{m}_\pm(x,t) = D_m\partial_x^2\delta\mathbf{m}_\pm + \mathbf{r}_\mathbf{m}\delta\mathbf{m}_\pm + \mathbf{r}_\mathbf{c}\delta\mathbf{c}|_{z=h}, \tag{11c}$$

[1] Linear stability analysis of vertically asymmetric steady states can be carried out analogously but is notationally more cumbersome because the linearization of bulk-surface coupling and membrane reactions is not identical on both membranes. Furthermore, for vertically asymmetric steady states, the eigenmodes don't decouple into vertically symmetric and antisymmetric bulk-modes.

where the matrices

$$\boldsymbol{f}_{\mathbf{c},\mathbf{m}} = \partial_{\mathbf{c},\mathbf{m}} \boldsymbol{f}\big|_{(\tilde{\mathbf{c}}_\mathrm{s},\tilde{\mathbf{m}}_\mathrm{s})}, \quad \mathbf{r}_{\mathbf{c},\mathbf{m}} = \partial_{\mathbf{c},\mathbf{m}} \mathbf{r}\big|_{(\tilde{\mathbf{c}}_\mathrm{s},\tilde{\mathbf{m}}_\mathrm{s})}, \tag{12}$$

are the linearized attachment-detachment kinetics and membrane reactions evaluated for the vertically symmetric steady state $(\widetilde{\mathbf{c}}_\mathrm{s}, \widetilde{\mathbf{m}}_\mathrm{s})$. For a LHSS, these coefficient matrices are constant in space. This enables us to find the spatial eigenmodes of the system analytically and reduce the set of linear PDEs (11a)–(11c) to an algebraic problem that then can be efficiently solved numerically.

**Spatial eigenmodes and finding their growth rates.** The key idea to solve the linearized dynamics Eqs. (11a)–(11c) is a separation of the time and space dependence in the form of *elementary perturbations*

$$\delta\mathbf{c}(x,z,t) = \Phi_c(x,z)\, e^{\sigma t}\, \delta\hat{\mathbf{c}}, \tag{13a}$$

$$\delta\mathbf{m}_\pm(x,t) = \Phi_m(x)\, e^{\sigma t}\, \delta\widehat{\mathbf{m}}_\pm, \tag{13b}$$

A general solution can then be constructed from a superposition of elementary perturbations. Our goal is to find the exponential growth rates $\sigma$. The growth rate with the largest real part determines the stability of the steady state. If it is positive, the steady state is *linearly unstable* because the corresponding elementary perturbation will grow exponentially in time. The spatial structure of this fastest growing elementary perturbation informs about the dynamics in the vicinity of the steady state.

For the separation ansatz Eq. (13) to be consistent, the elementary perturbations need to decouple from one another, that is, they must diagonalize all spatial differential operators *simultaneously*. This is the defining property of elementary perturbations. We denote the *spatial eigenmodes* of the diffusion operators on the membrane and in the bulk by $\Phi_m(x)$ and $\Phi_c(x,z)$ respectively. To construct elementary perturbations based on these eigenmodes, they need to diagonalize the bulk-surface coupling, meaning that they must fulfill the condition $\partial_z \Phi_c(x,z)|_{z=\pm h} \propto \Phi_m(x)$.

Because the geometry with flat membranes obeys the translational symmetry in $x-direction$ of the diffusion operators in the bulk and on the membranes, we can find the spatial eigenmodes by a separation of the spatial variables

$$\Phi_{c_i}(x,z) = Z_i(z)\Phi_m(x), \tag{14}$$

with $i = \mathrm{D}, \mathrm{DD}, \mathrm{E}$. Plugging this ansatz into the linear bulk dynamics, we obtain

$$(\sigma + \beta_i) Z_i(z)\Phi_m(x) = D_c Z_i(z)\Phi_m''(x) + D_c Z_i''(z)\Phi_m(x),$$

with $\beta_{\mathrm{D,E}} = 0$ and $\beta_{\mathrm{DE}} = \lambda$. Because there are no mixed spatial derivatives, we can rewrite this as

$$(\sigma + \beta_i) - D_c \frac{Z_i''(z)}{Z_i(z)} = \frac{\Phi_m''(x)}{\Phi_m(x)} = -q^2 = \mathrm{const},$$

where we introduced the lateral wavenumber $q$ in the separation constant, anticipating the solution

$$\Phi_m(x) = \cos(qx).$$

As required for consistency of the separation ansatz Eq. (14), this solution also diagonalizes diffusion operator $\partial_x^2$ on the membrane. For the vertical bulk profiles $Z_i(z)$ we have

$$0 = D_c Z_i''(z) - (\sigma + \beta_i + D_c q^2) Z_i(z).$$

Under the replacement $\lambda \to \sigma + \beta_i + D_c q^2$ this equation is equivalent to the equation for the bulk steady state profile of $c_{\mathrm{DD}}$, Eq. (8). Thus, we can immediately write down the symmetric and antisymmetric solutions

$$Z_i^{\mathrm{s}}(z;\sigma,q) = \frac{\cosh(\sqrt{q^2 + (\sigma+\beta_i)/D_c}\, z)}{\cosh(\sqrt{q^2 + (\sigma+\beta_i)/D_c}\, h)}, \tag{15a}$$

$$Z_i^{\mathrm{as}}(z;\sigma,q) = \frac{\sinh(\sqrt{q^2 + (\sigma+\beta_i)/D_c}\, z)}{\sinh(\sqrt{q^2 + (\sigma+\beta_i)/D_c}\, h)}. \tag{15b}$$

These symmetric and antisymmetric bulk profiles are orthogonal, i.e. they do not couple to one another. Hence, for each lateral wavenumber $q$, and the corresponding lateral mode $\Phi_m(x;q) = \cos(qx))$, there are two orthogonal bulk-eigenmodes, $\Phi_c^{\mathrm{s}}(x,z;q,\sigma) = \cos(qx)Z_i^{\mathrm{s}}(z;\sigma,q)$ and $\Phi_c^{\mathrm{as}}(x,z;q,\sigma) = \cos(qx)Z_i^{\mathrm{as}}(z;\sigma,q)$.

These two eigenmodes correspond to the two vertical transport modes discussed in the main text: membrane-to-membrane transport and membrane-to-bulk transport Note that in the limit of large bulk heights, they become equivalent since $\sinh(z)$ and $\cosh(z)$, which asymptotically approach $\exp(z)$ for large $z$. Since for linear stability, only the vertical bulk-gradient at the membrane surface matters, the stability properties of these modes become identical in the asymptotic limit $H \to \infty$; see SM.)

We can now insert these eigenmodes into the linearized bulk-surface coupling and membrane reactions, Eqs. (11b) and (11c), to obtain the linear set of equations

$$\underbrace{\begin{pmatrix} -D_c\,\mathbf{\Gamma}^{\mathrm{s,as}}(\sigma,q) + \boldsymbol{f}_{\mathbf{c}} & \boldsymbol{f}_{\mathbf{m}} \\ \mathbf{r}_{\mathbf{c}} & -\sigma - q^2 D_m + \mathbf{r}_{\mathbf{m}} \end{pmatrix}}_{=:\,\mathbf{M}_{\mathrm{s,as}}(\sigma,q)} \begin{pmatrix} \delta\hat{\mathbf{c}} \\ \delta\widehat{\mathbf{m}} \end{pmatrix} = 0, \tag{16}$$

with the coupling matrix $\mathbf{\Gamma}^{\mathrm{s,as}} := \mathrm{diag}(\Gamma_{\mathrm{D}}^{\mathrm{s,as}}, \Gamma_{\mathrm{DD}}^{\mathrm{s,as}}, \Gamma_{\mathrm{E}}^{\mathrm{s,as}})$, where

$$\Gamma_i^{\mathrm{s}} := \sqrt{q^2 + (\sigma+\beta_i)/D_c}\,\tanh\left(\sqrt{q^2 + (\sigma+\beta_i)/D_c}\,h\right), \tag{17a}$$

$$\Gamma_i^{\mathrm{as}} := \sqrt{q^2 + (\sigma+\beta_i)/D_c}\,\coth\left(\sqrt{q^2 + (\sigma+\beta_i)/D_c}\,h\right), \tag{17b}$$

for symmetric and antisymmetric vertical eigenmodes respectively.

The system of linear equations Eq. (16) has non-trivial solutions $(\delta\hat{\mathbf{c}}, \delta\widehat{\mathbf{m}})$ only when the determinant of the matrix $\mathbf{M}_{\mathrm{s,as}}(\sigma,q)$ vanishes, i.e. for pairs $(\sigma,q)$ that solve the characteristic equation $\det \mathbf{M}_{\mathrm{s,as}}(\sigma,q) = 0$. This determines a relationship between the lateral wavenumber $q$ and growth rates $\sigma_{\mathrm{s,as}}(q)$ defined by the complex solutions of

$$\sigma_{\mathrm{s}}(q):\ \det \mathbf{M}_{\mathrm{a}}\big(\sigma_{\mathrm{a}}(q), q\big) = 0, \tag{18}$$

$$\sigma_{\mathrm{as}}(q):\ \det \mathbf{M}_{\mathrm{as}}\big(\sigma_{\mathrm{as}}(q), q\big) = 0. \tag{19}$$

The relationship $\sigma(q)$ is called dispersion relation. As a result of the top-down symmetry (vertical parity symmetry) we have separate dispersion relations for vertically symmetric and antisymmetric perturbations. Note that there are infinitely many solutions $\sigma_{\mathrm{s,as}}(q)$ of Eqs. (18) and (19) for each wavenumber $q$. Therefore, the dispersion relations have infinitely many branches. Here, we are only interested in the branch with the largest real part for the symmetric and antisymmetric modes respectively.

To obtain the dispersion relations, we solve Eqs. (18) and (19) numerically using a Newton method (as provided by Mathematica's built-in function `FindRoot[]`). Representative examples of dispersion relations in different parameter regimes are shown in Supplementary Fig. 3.

**Limits of large bulk height and large wavenumbers.** There are two limits where the growth rates of the symmetric and the asymmetric modes coincide: (i) large bulk heights ($h \gg \sqrt{D_c/\lambda}$) and (ii) large lateral wavenumbers ($q^2 \gg \lambda/D_c$). In both limits, the bulk-surface coupling coefficients associated to the symmetric and antisymmetric bulk modes, Eqs. (17a) and (17b), asymptotically approach one another. This means that the distinction between symmetric and and antisymmetric perturbations becomes insignificant.

The physical reason for this is different for the two cases. In the case of large bulk height, the two membranes effectively decouple. In the case of large lateral wavenumbers, corresponding to short lateral wavelengths, lateral gradients dominate the dynamics of the perturbation such that the vertical gradients become irrelevant.

**Low bulk height limit.** In the limit of low bulk height, the antisymmetric coupling coefficient Γas becomes large. This suppresses instability of antisymmetric modes at low bulk heights.

## Supplementary Method 3. Numerical simulations and local equilibria analysis

We performed numerical simulations in COMSOL Multiphysics [8]. Crucially, this finite element based software can handle reaction–diffusion systems with bulk-surface coupling. We performed simulations in the full 2+3D geometry and in a reduced 1+2D geometry that represents a slice through the full geometry (see Sec.  above). Simulations in the full geometry are computationally very costly and were therefore only performed in comparatively small systems (200 µm edge length) for representative parameter sets. Simulations in large systems (500 µm length) over long timescales ($10^4$ s) were performed in the 1+2D slice geometry. The setup files for all simulations are included in the Data Repository. Note that the commercially available software COMSOL Multiphysics is required to open these files and run the simulations. Details on how the adiabatic sweeps of bulk height (cf. Fig. 4A–C) were performed are given in Sec. .0.3.

In addition to simulations in the box-shaped microchamber geometry, we performed simulations in cell geometry (cylinder with spherical caps); see Movies 19 and 20.

To generate snapshots, kymographs, and movies from 2+3D simulations (cf. Fig. 1 and Movie S3), we exported the membrane concentrations of MinD and MinE as density plots and overlaid them in green and red respectively using a Mathematica script. Similarly, we exported the MinD concentration at the top- and bottom membrane and overlaid them in blue and orange to show the in-phase and anti-phase synchronization, or lack thereof, between the two opposite membranes (cf. Fig. 4).

**Characterizing large amplitude patterns.** The patterns found in numerical simulations have large amplitudes, meaning they are far away from the homogeneous steady states around which we linearized the full set of nonlinear equations. As the amplitude of a pattern grows, nonlinearities will lead to coupling between different spatial modes. Therefore, the maintenance — and thus the properties — of patterns far away from the homogeneous steady state cannot be described by the linearized dynamics close to the homogeneous steady state (linear stability analysis). Hence, it is not clear a priori whether the oscillation modes identified by linear stability analysis the full set of nonlinear equations the qualitative features of large-amplitude patterns.

To study large-amplitude patterns, we make use of the fact that the total densities of MinD and MinE are conserved by the protein-protein interactions. We recently developed a theoretical framework for such mass-conserving reaction–diffusion systems that enables one to study the emergence and maintenance of large amplitude patterns [3, 9]. The core idea is to laterally dissect the system into notional compartments, and first study the dynamics in each compartment independently of its coupling to nearby compartments. In each isolated compartment, the local reactive equilibrium and its linear stability properties can be used as proxies to estimate the local dynamics. Importantly, these local dynamics depend on the total densities of MinD and MinE within the compartment. This dependence is captured by the phase diagram in the parameter space of total densities (see phase diagrams in Supplementary Fig. 4). The active oscillation modes encountered in an extended range of this phase diagram characterize the dynamics of large amplitude patterns.

As an example, compare the total density phase diagram at low bulk height to the one at large bulk height (Supplementary Fig. 4). In the former case, only lateral instability of the vertically symmetric mode occurs. Hence, only the lateral oscillation mode is active in this regime. In the case of larger bulk height, mass redistribution can trigger local vertical oscillations which have a substantial impact on pattern formation [3].

In the following we present several concrete examples, based on numerical simulations, showing the characterization of large amplitude patterns in terms of the local equilibria and their stability. In particular, we illustrate how mass redistribution locally (and regionally) activates oscillation modes that are not active at the homogeneous steady state.

We restrict the exemplary analysis to the low bulk-height regime and the large bulk height regime, corresponding to the *in vivo* setting and classical *in vitro* setups respectively. An extensive study of the dynamics in the large bulk height regime — with focus on the transition from chemical turbulence to order (SW/TW) — was performed in a previous theoretical study [3]. Analysis of the rich variety of patterns in the intermediate bulk-height regime where anti-phase oscillations between the two membranes play an important role is beyond the scope of this work.

This is an interesting avenue for future research.

Both, at low and large bulk heights, the simulations and local equilibria analysis can be performed in a geometry with membrane at only one boundary ($z = h$) and a reflective boundary opposite to it at $z = 0$. At low bulk heights, strong vertical membrane-to-membrane coupling leads to in-phase synchronization, so the system is always symmetric under vertical reflection $z \to -z$. At large bulk height, the two membrane decouple because the large bulk in-between them serves as a reservoir. In that case, the two membranes with the respective cytosol volume above/below can be considered separately by introducing a no-flux boundary at the center plane $z = 0$.

**Local equilibria analysis.** Local equilibria analysis starts with the full pattern dynamics obtained from a numerical simulation. From this data, one then calculates the instantaneous local masses (total densities) at each point on the membrane (on a grid sufficiently fine to resolves the patterns' structures).

In bulk-surface coupled systems with a vertically extended bulk, the local nonlinear reactions at the surface (membrane) crucially involve attachment and detachment processes that generate bulk fluxes in the vertical direction orthogonal to the membrane. To preserve these vertical gradients the dissection into compartments is performed only in the lateral direction. Hence, a local compartment comprises a membrane "point" with a extended cytosol column above it. This vertically extended bulk introduces a subtlety in the definition of the instantaneous local masses. Only the cytosolic density in the vicinity of the membrane participates in the nonlinear interactions at each point in time. Proteins further away from the membrane first need to diffuse to the membrane before they can interact with membrane-bound proteins. Therefore, only the cytosolic density at the membrane, not the density away from the membrane is taken into account to calculate the *instantaneous* local masses

$$n_\mathrm{D}^\mathrm{inst.}(x,t) := c_\mathrm{D}(x, z = h, t) + h^{-1}[m_\mathrm{d}(x,t) + m_\mathrm{de}(x,t)], \tag{20}$$

$$n_\mathrm{E}^\mathrm{inst.}(x,t) := c_\mathrm{E}(x, z = h, t) + h^{-1} m_\mathrm{de}(x,t). \tag{21}$$

This so called "adiabatic extrapolation" was introduced in the supplemental material of [3].

From the instantaneous local masses the local equilibria and their local and lateral stability can then be calculated. Alternatively, Plotting the local total densities in the $(n_\mathrm{D}, n_\mathrm{E}/n_\mathrm{D})$-phase diagram (see e.g. Supplementary Fig. 4) enables one to visually read off the local stability properties. The positions of the local equilibria (i.e. the equilibrium concentrations) and their stability properties serve as spatially and temporally resolved proxies for the instantaneous local dynamics. In particular, the instabilities reveal which oscillation modes are active at each point in space and time. These oscillation modes drive the formation of lateral gradients which, in turn, drive lateral mass redistribution that continually changes the instantaneous local masses.

This interplay between mass-redistribution, moving local equilibria, and their dynamically changing local and lateral stability governs the dynamics of the spatially extended systems. Movies 16–18 visualize this interplay for three examples: SW at low bulk height as well as TW and SWC at large bulk height. In each video, the three panels show MinD's membrane density profile (*top left*), the instantaneous total density profiles of MinD and MinE (green/red line, *bottom left*), and the instantaneous density distributions in the $(n_\mathrm{D}, n_\mathrm{E}/n_\mathrm{D})$-phase diagram (*top right*). Regions of local/lateral instability are shaded in orange/green in the membrane density plot and the phase diagram. Additionally, local equilibria shown as black dots in the membrane density plot. When the local equilibria are locally unstable, they are plotted as orange dots instead.

In summary, the local equilibria analysis reveals that standing waves at low bulk heights and at large bulk heights emerge by different mechanisms: At low bulk heights only lateral instability is involved, while at large bulk heights, both lateral and local instabilities play an important role.

## Supplementary Method 4. Adiabatic sweeps of the bulk height

To demonstrate multistability of qualitatively different patterns in the model, we show that the transitions between these states show hysteresis. To drive the system through the pattern

transitions, we adiabatically vary the bulk height in numerical simulations since this is the key parameter that separates qualitatively different dynamical regimes (cf. Fig. 2A).

**Numerical implementation.** A direct implementation of an adiabatically changing bulk height in numerical simulations would require a dynamically changing geometry and hence continuous updating of the mesh used in the finite element method. To circumvent this remeshing, we keep the geometry fixed and instead rescale the vertical coordinate, $z$, to emulate the adiabatically changing bulk height.

A geometry with dynamically changing bulk height $h(t)$ can be emulated in a geometry with fixed height $\hat{h}$ by the rescalings $z \to [h(t)/\hat{h}]\hat{z}$, $\partial_z \to [\hat{h}/h(t)]\partial_{\hat{z}}$, and $c_i \to [h(t)/\hat{h}]\hat{c}_i$ in the bulk dynamics Eq. (22), the bulk-surface coupling Eq. (23), and the total average density integrals Eq. (7). Note the due to these rescalings, the Laplace operator the coordinate frame of the fixed geometry becomes $\widehat{\nabla}^2 = \partial_x^2 + [\hat{h}/h(t)]^2 \partial_{\hat{z}}^2$. To absorb the rescaled $z$-coordinate in the parameters of the rescaled system, we need to allow for anisotropic bulk diffusion $\hat{D}_{c,x} \neq \hat{D}_{c,z}$, by in the bulk dynamics

$$\partial_t \hat{c}_i(x, \hat{z}, t) = D_{c,x}\partial_x^2 \hat{c}_i + \hat{D}_{c,z}\partial_{\hat{z}}^2 \hat{c}_i - \beta_i \hat{c}_i, \tag{22}$$

and the bulk-surface coupling

$$-\hat{D}_{c,z}\partial_{\hat{z}}\mathbf{c}|_{\hat{z}=\pm\hat{h}} = \pm \boldsymbol{f}(\mathbf{m}_\pm, \hat{\mathbf{c}}|_{\hat{z}=\pm h}), \tag{23}$$

With this, the parameters in the rescaled system read

$$\widehat{D}_{c,z} := \frac{\hat{h}^2}{h(t)^2} D_{c,z}, \quad \hat{k}_{\mathrm{D,dD,dE}} := \frac{\hat{h}}{h(t)} k_{\mathrm{D,dD,dE}}, \quad \hat{\bar{n}}_{\mathrm{D,E}} := \frac{h(t)}{\hat{h}} \bar{n}_{\mathrm{D,E}}. \tag{24}$$

(Note that the rescaling of the average total densities $\bar{n}_{\mathrm{D,E}}$ is required because we want to keep these quantities fixed while the dynamics conserves the absolute number of proteins $N_{\mathrm{D,E}} = h \cdot L \cdot \bar{n}_{\mathrm{D,E}}$.)

By simulating a system with the "hat" parameters and a fixed geometry of height $\hat{h}$ we emulate a system with the original parameters and a bulk height $h(t)$ that may change dynamically. This enables us to implement an adiabatic sweep (slow timescale $\tau = \varepsilon t$) of the system height $h(\tau)$ without remeshing by adiabatically changing the parameters according to Eq. (24). The diffusion constant $\widehat{D}_{c,z}(\tau)$ and the kinetic rates $\hat{k}_{\mathrm{D,dD,dE}}(\tau)$ appear explicitly in the dynamics and, hence, can be changed directly. In contrast, the average total densities $\hat{\bar{n}}_{\mathrm{D,E}}(\tau)$, enter implicitly via the conservation laws Eq. (7) and can not be changed directly. Instead, we implement their slow, rescaling-induced rate of change

$$\partial_\tau \hat{\bar{n}}_{\mathrm{D,E}}(\tau) = \frac{\partial_\tau h(\tau)}{\hat{h}} \bar{n}_{\mathrm{D,E}}$$

by effective global production/degradation terms in the cytosol

$$\partial_t \hat{c}_i(x, \hat{z}, t) = D_{c,x}\partial_x^2 \hat{c}_i + \widehat{D}_{c,z}\partial_{\hat{z}}^2 \hat{c}_i - \beta_i \hat{c}_i + \frac{\partial_\tau h(\tau)}{\hat{h}} \bar{n}_i, \quad i = \mathrm{D, E}. \tag{25}$$

The additional term $\propto \partial_\tau h(\tau) \sim \mathcal{O}(\varepsilon)$ is negligible on the fast timescale while it ensures the correct scaling of the total average densities (cf. Eq. (24)) on the slow timescale where the emulated bulk height changes.

We performed the adiabatic sweeps of the bulk height between 2 µm and 20 µm and on a domain of length $L = 100\,\mu\mathrm{m}$ with periodic boundaries. This small system size stabilizes the anti-phase membrane to membrane oscillations against the formation of phase defects that leads to defect-mediated turbulence. The average MinD and MinE masses were set to $\bar{n}_{\mathrm{D}} = 400\,\mu\mathrm{m}^{-3}$, $\bar{n}_{\mathrm{E}} = 290\,\mu\mathrm{m}^{-3}$. All other parameters are given in Supplementary Table 1.

**Transitions from anti-phase to in-phase synchronized patterns.** In the main text (Fig. 4), we show the transition from in-phase synchronized standing waves (SW) to anti-phase synchronized standing/traveling waves (SW/TW) and from large-scale anti-phase oscillations (OSC) to anti-phase synchronized SW/TW. Because these transitions happen rapidly within a few periods we could directly visualize them in kymographs.

**Transitions from anti-phase to in-phase synchronized patterns.** The transitions from anti-phase synchronized OSC/SW/TW to in-phase synchronized SW happen much more slowly which makes it hard to visualize them in kymographs. Instead, we quantify the salient features of these patterns that clearly discriminate the different pattern types. Time series of these quantities show the pattern transitions (Supplementary Fig. 8).

To discriminate between in-phase and anti-phase synchronization between the top and bottom membrane, we calculate the correlation between the membrane concentrations in a moving window of 30 s length. The correlation is close to 1 for in-phase synchronized patterns and close to $-1$ for anti-phase synchronized patterns/oscillations.

In addition, we calculate the Fourier amplitudes of the spatial concentration profile for each time point. For homogeneous oscillations, only the zero mode, $\tilde{u}_0$, has non-zero amplitude. To quantify the pattern amplitude of the SW, we use the amplitude of the dominant Fourier mode $\tilde{u}_3$, which, in the domain of length $L = 100\,\mu\text{m}$ turns out to be the third mode corresponds to a wavelength of approx. 33 µm.

Because the patterns are oscillatory (modulated SW/TW) the Fourier mode amplitudes oscillate. The oscillation period is on the order of magnitude of 10 s, much shorter than the timescale of 1000 s on which the transition between the patterns take place. We therefore plot the envelope of the oscillating mode amplitudes. The envelope is obtained by finding the minimum and maximum in a sliding window of width 30 s.

**OSC to in-phase SW.** To demonstrate the transition from OSC to in-phase SW (Supplementary Fig. 8A), we initialized a system of bulk height $H = 8\,\mu\text{m}$ with homogeneous membrane-to-membrane oscillations (these oscillation can be induced by starting the system with a strong asymmetry in the protein concentrations between top and bottom membrane). The bulk height is then gradually reduced at a rate of $6 \times 10^{-4}\,\mu\text{m}\,\text{s}^{-1}$. The envelope plot of the laterally homogeneous mode $\tilde{u}_0$ shows that homogeneous oscillations persist up to approx. 6000 s. After the homogeneous oscillations have decayed, the system very is close to the homogeneous steady state. Hence, the initial amplitude from which in-phase synchronized SW patterns start to grow is very small, and the emerges by slow exponential growth. In contrast to the slow, gradual transition of the oscillation and pattern amplitude, the top-bottom correlation changes abruptly, clearly marking the transition from anti-phase to in-phase synchronization.

Note that, as we showed in the main text (cf. Fig. 4), starting from in-phase SW at low bulk height and slowly increasing bulk height, the in-phase pattern persist up to a bulk height of 12 µm. Hence the regime of multistability between anti-phase OSC and in-phase SW ranges from 6 to 12 µm, as show in Fig. 4 in the main text.

**Anti-phase to in-phase waves.** To demonstrate the transition from anti-phase SW/TW to in-phase SW (shown in Supplementary Fig. 8B), we initialized a system of bulk height $H = 20\,\mu\text{m}$ at the homogeneous steady state with a small random perturbation. At this bulk height, the system exhibits anti-phase TW/SWs. Upon reducing the bulk height at a rate of $8 \times 10^{-4}\,\mu\text{m}\,\text{s}^{-1}$, these waves persist down to $H \approx 6\,\mu\text{m}$. As for the OSC to in-phase SW transition, the pattern amplitude decays to almost zero, before in-phase SW start growing. In contrast, the transition from in-phase to anti-phase synchronized waves/oscillations for increasing bulk height takes place "directly", without transiently going through a nearly homogeneous state (see Fig. 4).

## Supplementary Discussion 1. Experimental phase diagram

In this section, we describe and quantify the patterns observed in the different regions in the experimental phase diagram (Fig. 3 in the main text).

**Traveling waves.** As predicted by the model, traveling waves are found mainly for large bulk heights ($> 15\,\mu$m) and sufficiently large E:D ratios ($\gtrsim 1$). In addition, we found in traveling waves down to $2\,\mu$m bulk height at an E:D ratio of 2. We quantified the experimentally observed patterns using autocorrelation analysis (Fig. S9). Traveling waves exhibit oscillation periods that increase as a function of E:D ratio and bulk height in the range 2.5 to 8 min. Their wavelength ranged mostly from 35 to $55\,\mu$m and did not vary systematically across the conditions we investigated.

**Standing wave chaos.** Standing wave chaos occurs in two distinct regions of the phase diagram. First, for low bulk heights, where our theoretical analysis shows that only lateral instability exists (cf. Fig. 2 and Supplementary Fig. 6). Second, for large bulk heights, but only at low E:D ratios, as was theoretically predicted in a previous study [3]. Similarly as for the traveling waves, we found that the oscillation period of standing waves varied over a broad range (from 4 to 37 min) increasing as a function of bulk height and E:D ratio. The wavelength of standing waves is approximately $40\,\mu$m, showing a slight increase as a function of bulk height. In addition to wavelength and oscillation frequency, we also quantified the width of fronts (also called "interfaces" or "domain walls") that connect low-density to high-density regions of the standing wave patterns (see Supplementary Fig. 11). The front width *in vitro* is around $5\,\mu$m, which is indeed quite close to the one of *in vivo* patterns ($\sim$1–$5\,\mu$m). Because the front width is directly determined by the lateral mode underlying pattern formation (see Ref. [9] and Supplementary Fig. 12 and Supplementary Discussion 2, this provides an additional link between the *in vivo* and *in vitro* dynamics.

**Homogeneous oscillations, phase waves, and defect-mediated turbulence.** Spatially large-scale oscillation phenomena commonly found in oscillatory media — homogeneous oscillations, phase waves, and defect-mediated turbulence — were found only for intermediate heights. They dominated for the low E:D ratios in particular, as also predicted by linear stability analysis for the mathematical model (cf. Fig. 2A, note the small regime around $H = 10\,\mu$m, at the lower edge of the regime of instability where only the local membrane-to-membrane oscillation mode is unstable.). We observed robust, homogeneous oscillations down to $8\,\mu$m bulk height (and one instance for $6\,\mu$m), indicating the critical bulk height for the onset of membrane-to-membrane oscillations in the experiment. This value is in good agreement with the minimal model where it is about $5\,\mu$m for the parameters used in this study. By autocorrelation analysis, we found oscillation periods between 4 min and 30 min where the period increases as a function of bulk height.

**Segmented waves ("amoebas").** In addition to the three pattern types presented in Figure 1, we also found a fourth type of pattern that resembles "segmented waves" [10, 11]. These patterns are similar to a phenomenon previously observed in the *in vitro* Min system where they were called "amoeba patterns" [6, 12]. The segmented waves consist of small separate "blobs" of MinD which, in contrast to standing waves, are not surrounded by MinE but instead feature an unidirectional MinE gradient, resulting in directional propagation of the blobs (Movie S14). This type of pattern occurred mostly at large E:D ratio ($\gtrsim 2$) and small to intermediate bulk heights (2–$25\,\mu$m). Due to its incoherent and non-oscillatory character, we did not characterize this pattern by autocorrelation analysis.

In the vicinity to the traveling wave regime, the segmented waves emerge due to the segmentation of the spiral wave front. Such "segmented spirals" were previously observed and studied in the BZ-AOT system [10, 11]. This might provide hints towards the mechanism underlying segmented wave formation. Explaining this phenomenon likely requires an extension of the minimal Min model, e.g. by the cytosolic switching dynamics of MinE [6].

## Supplementary Discussion 2. Nonlinear length-scale selection

In this section, we provide an extended discussion on the question of length-scale selection. In particular, we distinguish the pattern wavelength and the front width.

From a theoretical standpoint, the principles underlying nonlinear wavelength selection of large-amplitude patterns are largely unknown. As of yet, only (quasi-)stationary patterns in one- and two-variable systems have been systematically studied [13–15]. Importantly, the wavelength of strongly nonlinear patterns is not necessarily determined by the dominant linear instability of the homogeneous steady state. Instead, the wavelength is subject to a subtle interplay of local reactions and lateral transport (many nonlinearly coupled modes) and therefore can depend sensibly and non-trivially on parameters. For instance, a previous theoretical study of the *in vivo* Min system showed that a subtle interplay of recruitment, cytosolic diffusion and nucleotide exchange can give rise to "canalized transfer" which plays an important role for wavelength selection in this system [2]. Moreover, experimental studies on the reconstituted Min system showed that many "microscopic" details, like the ionic strength of the aqueous medium, temperature, and membrane charge can also affect the wavelength of Min patterns [16, 17].

Matching the wavelengths found in simulations to those found in experiments would require fitting of parameters, and, potentially, model extensions (e.g. the switching of MinE between active and inactive conformations [6]). The insights into the pattern-forming mechanisms presented here may aid such parameter calibration in future studies. However, because the principles underlying wavelength selection of fully developed nonlinear patterns are — on a general level — not understood yet, this would require running a large number of costly numerical simulations, which makes this task unfeasible with present day computers. (The simulations shown in Fig. 1D alone took over one month on a HPC workstation.) Moreover, it is not clear whether the same kinetic rates should be used for in *in vivo* and *in vitro* conditions, because the protein densities on the membrane are up to two orders of magnitude larger in the latter condition [18].

**Front width of standing waves.** Besides their wavelength, i.e. the distance between consecutive wave nodes, patterns have a second characteristic length-scale — the width of fronts, also called "interfaces" or "domain walls", that connect low-density to high-density regions. Local equilibria theory proposes that regional properties of fully developed, nonlinear patterns can be determined from a linearization of the dynamics in spatial regions [9, 19]. Specifically, for two-component mass-conserving reaction–diffusion systems, it has been shown that the front width is determined by the marginal mode (right edge of the band of unstable modes, marked by $q_{\mathrm{max}}$ in Supplementary Fig. 12) of the dynamics linearized around the inflection point [9]. Put heuristically, the lateral instability maintains ("spans") the steady state interface at the marginally stable length scale. This relationship ties the a characteristic length scale of the pattern (front width) to the mechanism underlying pattern formation (lateral instability). We hypothesize that this relationship carries over to mass-conserving reaction–diffusion systems with more than two components, like the Min system. To test this hypothesis we compare the front width measured in numerical simulations to the prediction from regional linear stability analysis (see Supplementary Fig. 12). The front width in the numerical simulations is measured as the distance between maxima and minima on either side of inflection points in the $m_{\mathrm{d}}$ profile. Front widths and amplitude (concentration difference between minimum and maximum) are measured for all fronts in a series of snapshots over several oscillation cycles of the standing wave.

Because the total densities at the patterns inflection points fluctuate around the average total densities $\bar{n}_{\mathrm{D}}, \bar{n}_{\mathrm{E}}$, we use these average densities to calculate the dispersion relation from which we predict the interface width as $\pi/q_{\mathrm{max}}$.

We vary the cytosol diffusion constant $D_c$ as a control parameter to demonstrate how the front width decreases with $D_c$. In cells, the cytosol diffusion constant is on the order of $10\,\mu\mathrm{m}^2\,\mathrm{s}^{-1}$ [4], compared to around $60\,\mu\mathrm{m}^2\,\mathrm{s}^{-1}$ in the reconstituted system [5]. This may explain why the front width observed *in vivo* is smaller than the front widths we measured for standing waves in low-height microchambers.

## Supplementary Figures

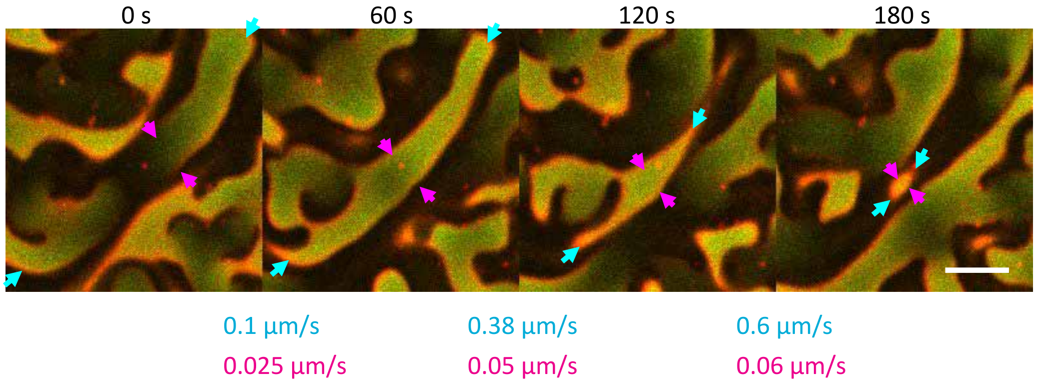


**Supplementary Figure 1. Diverse and dynamically changing wavefront propagation speeds in an experimentally observed chaotic standing wave pattern**. Cyan arrows indicate fast moving fronts while magenta arrows indicate slow moving fronts. In both cases velocity of wavefronts increases as the pattern edges get closer. ($H = 6\,\mu\mathrm{m}$, $1\,\mu\mathrm{M}$ MinD, $1\,\mu\mathrm{M}$ MinE; Scale bar: $30\,\mu\mathrm{m}$)

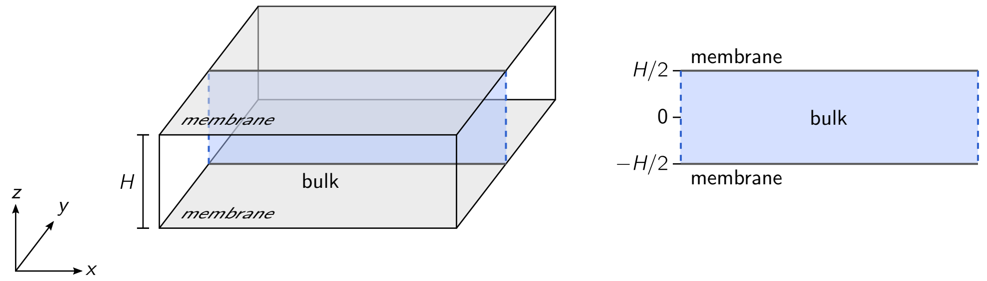


**Supplementary Figure 2. Domain geometries used for linear stability analysis and numerical simulations of the Min skeleton model.** (*Left*) Full three-dimensional "box geometry". (*Right*) Reduced two-dimensional geometry representing a slice through the full geometry.

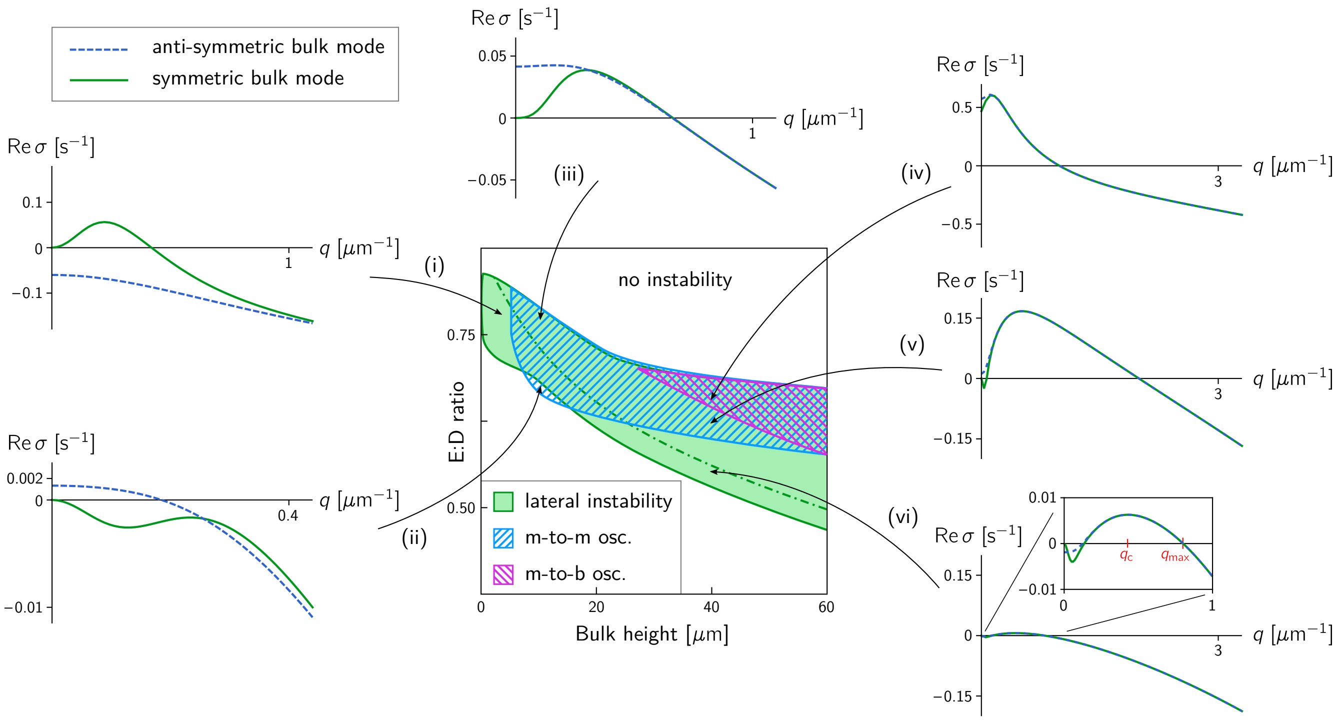


**Supplementary Figure 3. Representative dispersion relations in different regions of the phase diagram.** Each dispersion relation shows the real part of the growth rate $\sigma$ as a function of the lateral wavenumber $q$. The dispersion relations of the two vertical modes are shown as a solid green line (vertically symmetric) and a dashed blue line (vertically antisymmetric). The growth rate at $q = 0$ indicates stability against perturbations that do not involve lateral transport, that is, the homogeneous stability of a homogeneous steady state and the local stability of a laterally isolated compartment. (i) Lateral instability only for the vertically symmetric bulk mode and no local instabilities. This indicates that the lateral mode alone drives the dynamics. (ii) No instability for the vertically symmetric bulk mode and *local* instability of the vertically antisymmetric mode, indicating that only the vertical membrane-to-membrane mode drives the dynamics. (iii) Lateral instability of the symmetric bulk mode and local instability of the antisymmetric bulk mode, indicating that the lateral mode and the vertical membrane-to-membrane mode contribute to the dynamics. (iv) Local and lateral instability in both bulk modes, indicating that the lateral mode and the vertical membrane-to-bulk mode contribute to the dynamics. (v) Commensurable lateral instability i.e. $q_{\max} > 2q_{\mathrm{c}}$(see [3]). (vi) Incommensurable lateral instability, i.e. $q_{\max} < 2q_{\mathrm{c}}$ (see [3]). The regimes marked in the phase diagram are defined as follows. Lateral instability: $\max_q \operatorname{Re}\sigma_{\mathrm{s}} > 0$; membrane-to-membrane oscillatory local instability (m-to-m mode): $\operatorname{Re}\sigma_{\mathrm{as}}(q=0) > 0$; membrane-to-bulk oscillatory local instability (m-to-b mode): $\operatorname{Re}\sigma_{\mathrm{s}}(q=0) > 0$.

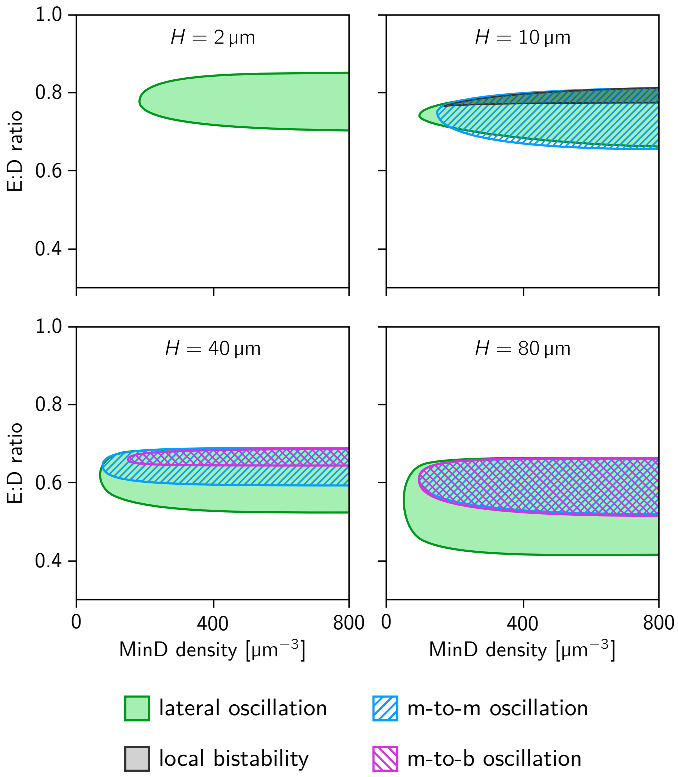


**Supplementary Figure 4. Phase diagrams in the parameter space of total MinD density ($\bar{n}_{\mathrm{D}}$) and the E:D ratio ($\bar{n}_{\mathrm{E}}/\bar{n}_{\mathrm{D}}$) for different bulk heights $H$.** For too low MinD density, the nonlinear feedback due to self-recruitment of MinD is too weak to drive instabilities. For sufficiently high MinD density, only the E:D ratio is important because the dynamics are driven by the competition of MinD self-recruitment to the membrane and MinE-driven MinD-detachment from the membrane. Throughout the paper, a value of $400\,\mu\mathrm{m}^{-3}$ for the average MinD density was used in the numerical simulations and linear stability analysis.

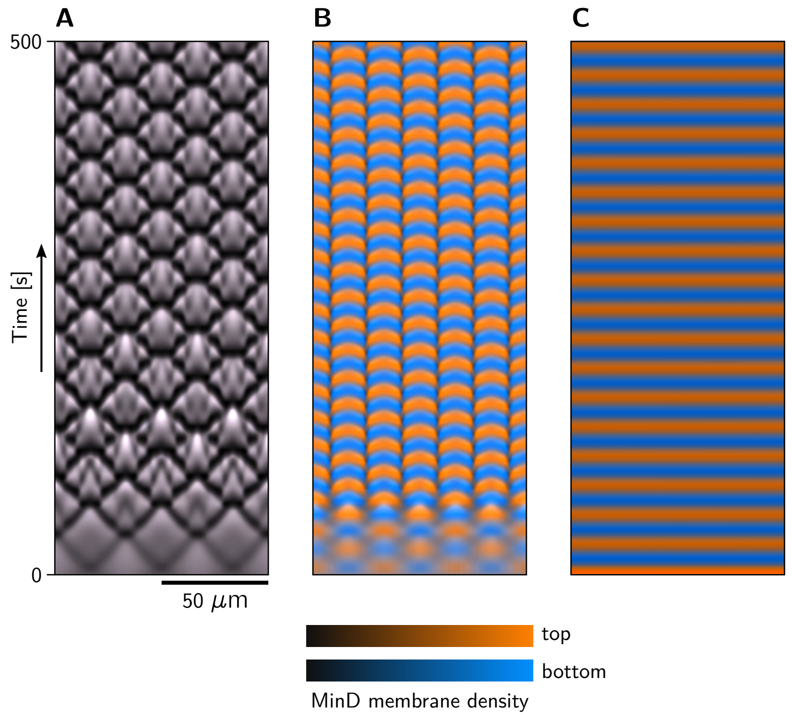


**Supplementary Figure 5. Pattern multistability at intermediate bulk height.** Simulations with identical parameters end up in different steady states depending on the initial perturbation. (**A**) Sinousoidal initial perturbation with the same phase on top and bottom membrane. The patterns on the top and bottom membrane are in perfect synchrony such that high density regions appear white in the displayed overlay as additive mixing of blue and orange yields white. (**B**) Sinusoidal initial perturbation with opposite phase on top and bottom membrane. (**C**) Entire mass initialized homogeneously on the top membrane. The periodic alternation of blue and orange in (B) and (C) indicates anti-phase synchronized patterns. (Simulation performed in 1+2D slice geometry, parameters: $H = 7\,\mu\text{m}$, $\bar{n}_\text{E}/\bar{n}_\text{D} = 0.725$, in each case 10% noise was added to the initial condition.)

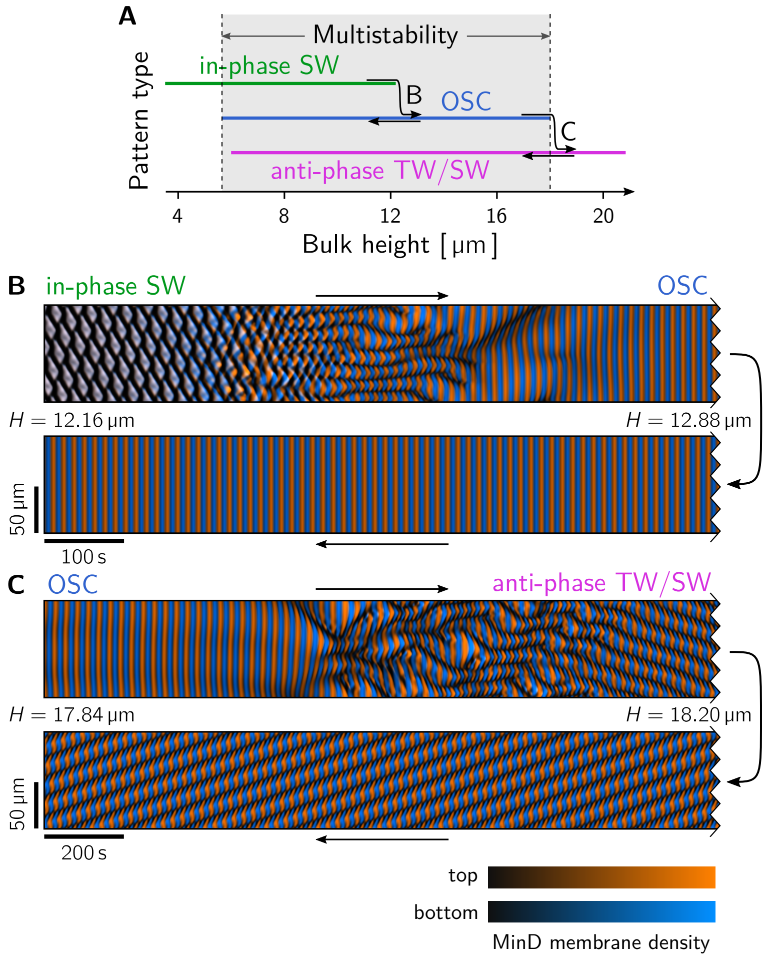


**Supplementary Figure 6. Hysteresis indicates multistability of different pattern types in numerical simulations.** (**A**) Using adiabatic parameter sweeps of the bulk height (along the red line, E/D = 0.725, in Fig. 2D in the main text) we demonstrate multistability of different pattern types. A hallmark of multistability is hysteresis as shown in (B) for the transition from in-phase standing waves (SW) to homogeneous oscillations (OSC); and in (C) for the transition to homogeneous oscillations to anti-phase traveling/standing waves (TW/SW). (**B**) Kymograph showing the transitions from in-phase SW to anti-phase OSC as the bulk height is adiabatically increased from 12.16 µm to 12.88 µm. Upon decreasing the bulk height back to 12.16 µm, the homogeneous oscillations persist. In fact the transition back to in-phase SW takes place around $H = 6$ µm, see Supplementary Fig. 8A. As in Supplementary Fig. 5, blue and orange mix to white in high density regions where the patterns are in-phase synchronized. (**C**) Kymograph showing the transitions from anti-phase OSC to anti-phase TW/SW as the bulk height is adiabatically increased from 17.84 µm to 18.20 µm. Upon decreasing the bulk height back to 17.84 µm, the anti-phase TW/SW pattern persists. Similarly as OSC, these patterns persist down to around $H = 6$ µm, see Supplementary Fig. 8B. (Simulations performed in 1+2D slice geometry; for details on the adiabatic sweeps see SI text.)

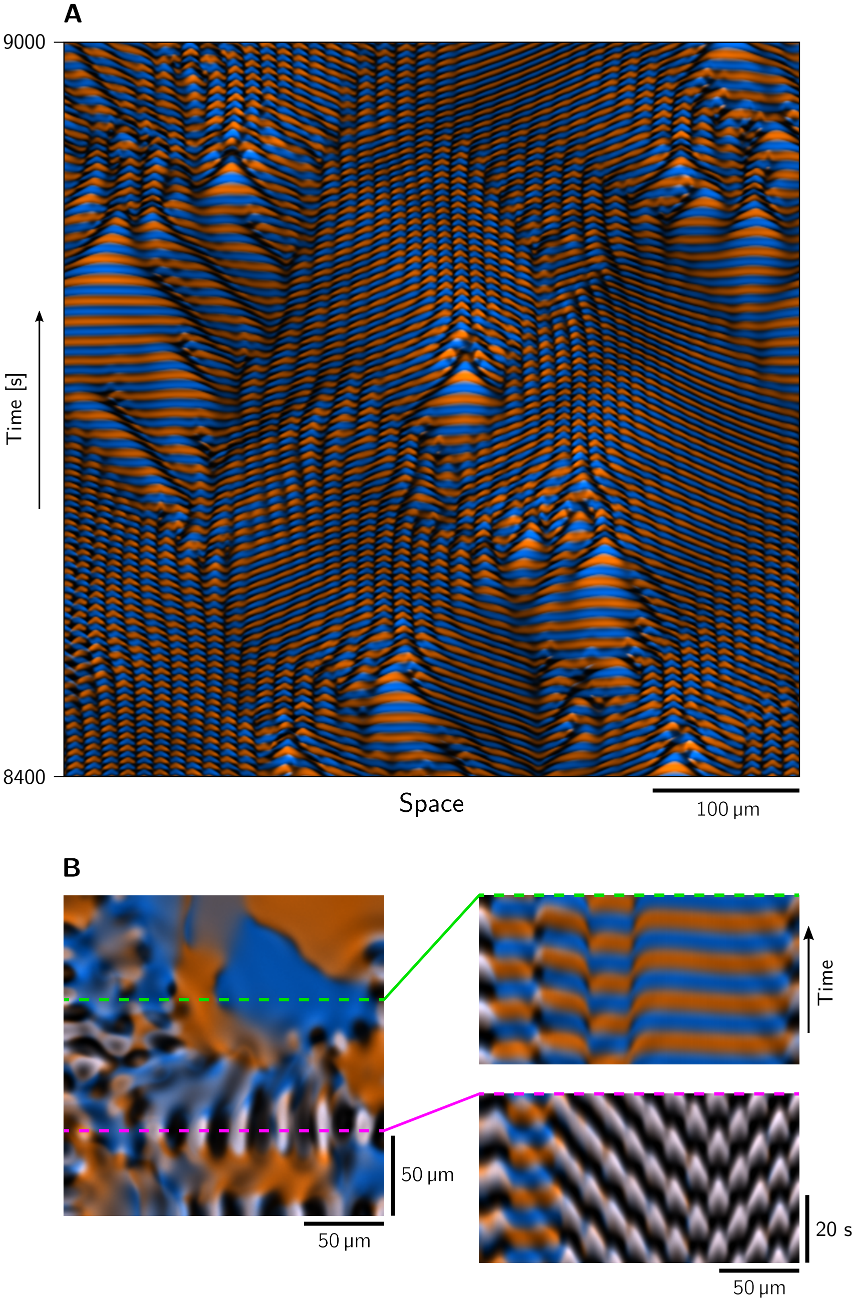


**Supplementary Figure 7. Coexistence of different pattern types in neighboring spatial regions.** (**A**) Kymograph from a simulation in 1+2D slice geometry showing spatiotemporal intermittency, i.e. coexistence of OSC, SW and TW in neighboring spatial regions with continual transitions between pattern types over time. (Parameters: $H = 18\,\mu\text{m}$, $\bar{n}_\text{E}/\bar{n}_\text{D} = 0.725$.) (**B**) Snapshot (left) and kymographs (right) from a in a simulation in full 2+3D box geometry showing coexistence of in-phase synchronized standing wave and anti-phase synchronized large-scale oscillations (see also Movie 10). As in Supplementary Fig. 5, blue and orange mix to white in high density regions where the patterns are in-phase synchronized. (Parameters: $H = 8\,\mu\text{m}$, $\bar{n}_\text{E}/\bar{n}_\text{D} = 0.7625$.)

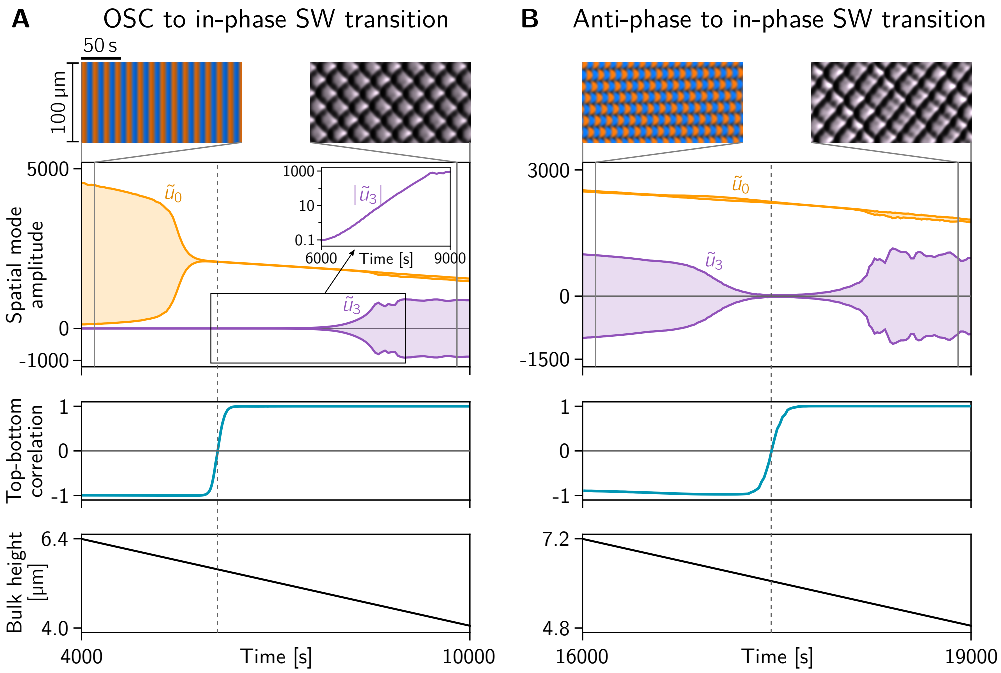


**Supplementary Figure 8. Transitions from anti-phase to in-phase synchronized patterns in adiabatic sweeps of the bulk height.** The kymographs show the first and last 200 s of each simulation respectively. (**A**) Transition from homogeneous anti-phase oscillations (OSC) to a standing wave (SW) pattern that is perfectly synchronized between the two membranes. The amplitude of the homogeneous mode $\tilde{u}_0(t)$ and the dominant Fourier mode $\tilde{u}_3(t)$ are shown as envelope plots in yellow and purple respectively. Inset: logarithmic plot of $|\tilde{u}_3|$, to highlight the slow exponential growth of the pattern after the homogeneous oscillations have vanished. The top-bottom correlation (teal line) transitions abruptly at $t \approx 6100$ s, corresponding to $H \approx 5.12$ µm. As in Supplementary Fig. 5, blue and orange mix to white in high density regions where the patterns are in-phase synchronized. (**B**) Transition from anti-phase synchronized SW/TW to in-phase SW. Note that the pattern amplitude, quantified by the amplitude of the dominant spatial Fourier mode $|\tilde{u}_3|$, goes down to almost zero, coming very close to the homogeneous steady state, at the point where the pattern synchronization switches from anti-phase to in-phase synchrony (see top-bottom correlation). The in-phase SW then grow from the nearly homogeneous transition state. (Simulation performed in 1+2D slice geometry, parameters: $H = 7$ µm, $\bar{n}_\mathrm{E}/\bar{n}_\mathrm{D} = 0.725$, in each case 10% noise was added to the initial condition.)

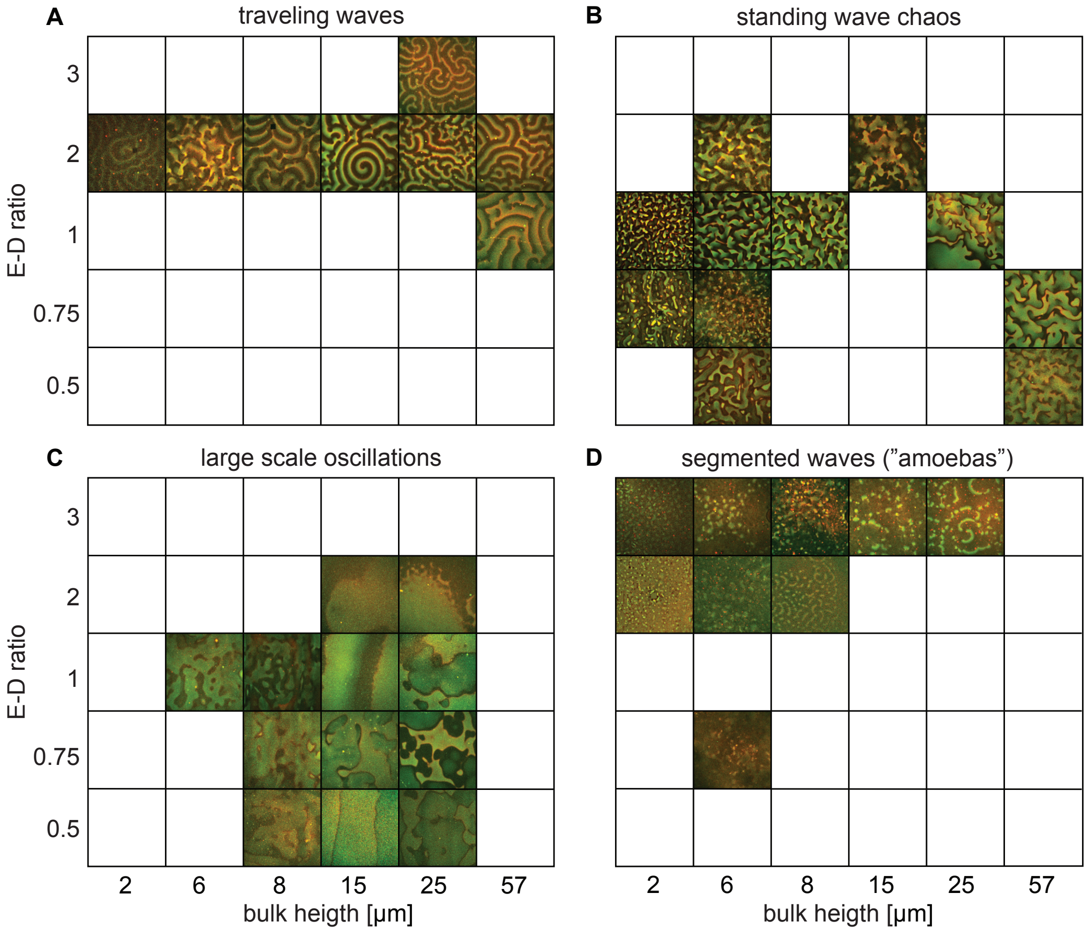


**Supplementary Figure 9.** Representative snapshots indicate where each of the four pattern types — traveling waves (TW), standing wave chaos (SWC), large scale oscillations (OSC) and segmented waves — was observed as a function of E:D ratio and bulk height (cf. Movies S11–S14 and Fig. 3 in the main text). Each snapshot shows an overlay of the MinD channel (green) and the MinE channel (red); field of view: 307 µm × 307 µm.

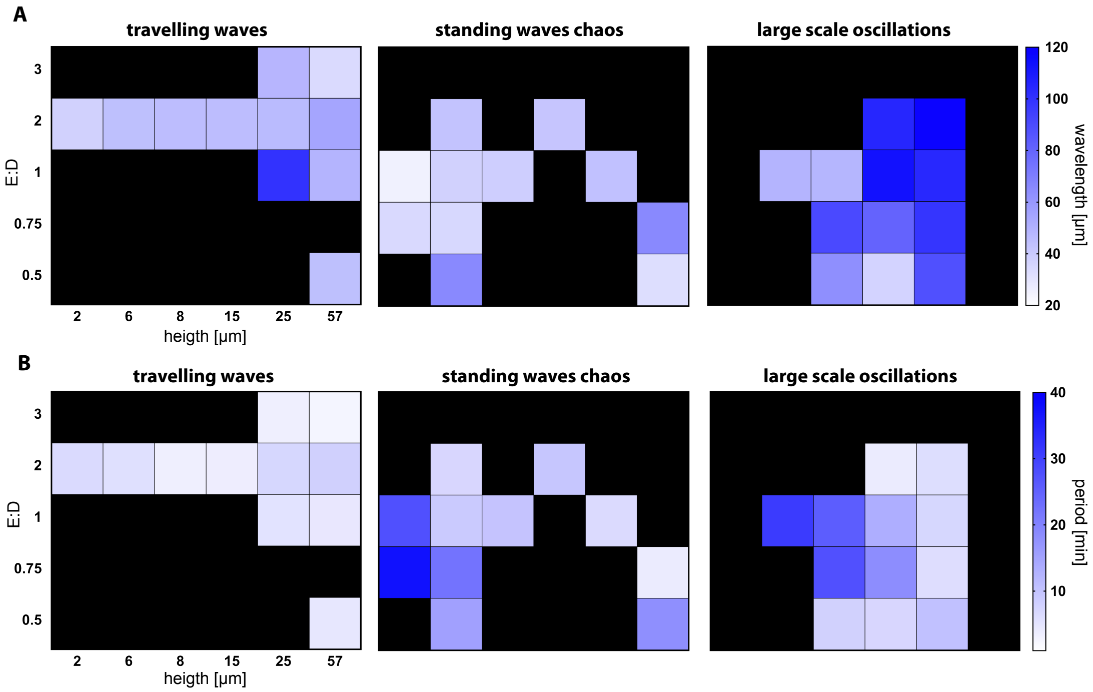


**Supplementary Figure 10. Quantification of the patterns as a function of bulk height and E:D ratio.** Characteristic wavelength (**A**) and oscillation period (**B**) were obtained by correlation analysis, as described in the Materials and Methods section. Black indicates conditions where the respective pattern type was not observed. (Source data are provided as a Source Data file.)

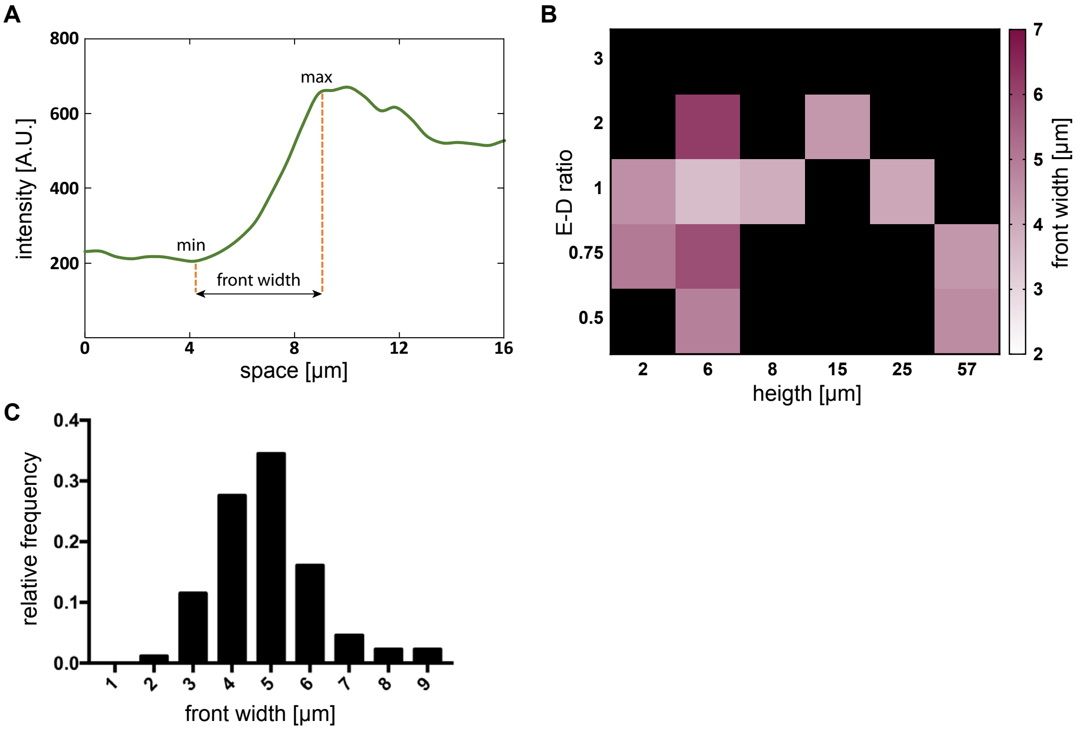


**Supplementary Figure 11. Front widths of chaotic standing waves found in experiments.** (**A**) Example of MinE intensity profile of a chaotic standing wave. Single arrows indicate points at minimum and maximum of a wave intensity. The double arrow (FW) indicates the distance between minimum and maximum measured as a front width. Minimum, maximum and distance between them were measured using a Matlab script. (**B**) Front widths of chaotic standing waves formed for different E:D ratios and bulk heights (for each parameter combination, the front width measured at $n > 20$ different spatial positions in a snapshot and then averaged). (**C**) Histogram of the front widths of chaotic standing waves across all tested bulk heights and E:D ratios (histogram over 81 individual width measurements). The average front width is 4.4 µm. (Source data are provided as a Source Data file.)

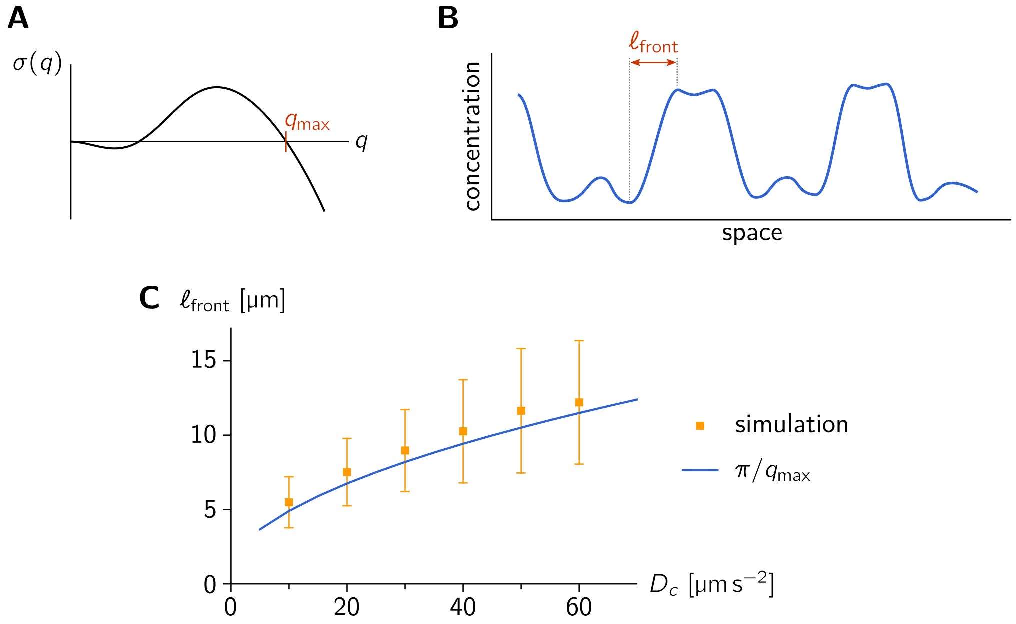


**Supplementary Figure 12. The marginal mode in the dispersion relation predicts the front width.** (**A**) Example of a dispersion relation with the marginal mode (right edge of the band of unstable modes) $q_{\max}$ marked. (**B**) Illustration of the front width as distance between consecutive maxima and minima of the (membrane) concentration profile of a fully developed nonlinear pattern. (**C**) Front width as a function of cytosol diffusion. Symbols: Front width obtained from numerical simulations averaged over all fronts identified in the $m_{\mathrm{de}}$ concentration profiles for several oscillation cycles. The average is weighted with the front amplitude. (We analyzed concentration profiles for 200 timepoints, amounting to a total of at least 7000 interface width measurements for each value of $D_c$. Symbols and error bars show the mean and one standard deviation, respectively. Source data are provided as a Source Data file.) Line: Front width predicted from marginal mode in the dispersion relation for $(n_{\mathrm{D}}, n_{\mathrm{E}}) = (\bar{n}_{\mathrm{D}}, \bar{n}_{\mathrm{E}})$. (Parameters: $H = 1\,\mu\mathrm{m}$, $\bar{n}_{\mathrm{E}}/\bar{n}_{\mathrm{D}} = 0.8$, system size $500\,\mu\mathrm{m}$; for remaining parameters see Supplementary Table 1.)

## Supplementary References


1. K. C. Huang, Y. Meir, and N. S. Wingreen. Dynamic structures in Escherichia coli: Spontaneous formation of MinE rings and MinD polar zones. *Proceedings of the National Academy of Sciences*, 100(22):12724–12728, 2003. ISSN 0027-8424, 1091-6490. doi: 10.1073/pnas.2135445100.

2. J. Halatek and E. Frey. Highly Canalized MinD Transfer and MinE Sequestration Explain the Origin of Robust MinCDE-Protein Dynamics. *Cell Reports*, 1(6):741–752, 2012. ISSN 22111247. doi: 10.1016/j.celrep.2012.04.005.

3. J. Halatek and E. Frey. Rethinking pattern formation in reaction–diffusion systems. *Nature Physics*, 14(5):507, 2018. ISSN 1745-2481. doi: 10.1038/s41567-017-0040-5.

4. G. Meacci, J. Ries, E. Fischer-Friedrich, N. Kahya, P. Schwille, and K. Kruse. Mobility of Min-proteins in *Escherichia coli* measured by fluorescence correlation spectroscopy. *Physical Biology*, 3(4):255–263, 2006. ISSN 1478-3975. doi: 10.1088/1478-3975/3/4/003.

5. M. Loose, E. Fischer-Friedrich, J. Ries, K. Kruse, and P. Schwille. Spatial Regulators for Bacterial Cell Division Self-Organize into Surface Waves in Vitro. *Science*, 320(5877): 789–792, 2008. ISSN 0036-8075, 1095-9203. doi: 10.1126/science.1154413.

6. J. Denk, S. Kretschmer, J. Halatek, C. Hartl, P. Schwille, and E. Frey. MinE conformational switching confers robustness on self-organized Min protein patterns. *Proceedings of the National Academy of Sciences*, 115(18):4553–4558, 2018. ISSN 0027-8424, 1091-6490. doi: 10.1073/pnas.1719801115.

7. Mathematica. Wolfram Research, Inc., 2019.

8. COMSOL Multiphysics. COMSOL AB, 2019.

9. F. Brauns, J. Halatek, and E. Frey. Phase-Space Geometry of Mass-Conserving Reaction-Diffusion Dynamics. *Physical Review X*, 10(4):041036, 2020. ISSN 2160-3308. doi: 10.1103/PhysRevX.10.041036.

10. V. K. Vanag and I. R. Epstein. Segmented spiral waves in a reaction-diffusion system. *Proceedings of the National Academy of Sciences*, 100(25):14635–14638, 2003. ISSN 0027-8424, 1091-6490. doi: 10.1073/pnas.2534816100.

11. I. R. Epstein and J. A. Pojman. *An Introduction to Nonlinear Chemical Dynamics: Oscillations, Waves, Patterns, and Chaos*. Topics in Physical Chemistry. Oxford University Press, New York, 1998. ISBN 978-0-19-509670-5.

12. A. G. Vecchiarelli, M. Li, M. Mizuuchi, L. C. Hwang, Y. Seol, K. C. Neuman, and K. Mizuuchi. Membrane-bound MinDE complex acts as a toggle switch that drives Min oscillation coupled to cytoplasmic depletion of MinD. *Proceedings of the National Academy of Sciences*, 113(11):E1479–E1488, 2016. ISSN 0027-8424, 1091-6490. doi: 10.1073/pnas.1600644113.

13. P. Politi and C. Misbah. When Does Coarsening Occur in the Dynamics of One-Dimensional Fronts? *Physical Review Letters*, 92(9):090601, 2004. ISSN 0031-9007, 1079-7114. doi: 10.1103/PhysRevLett.92.090601.

14. T. Kolokolnikov, T. Erneux, and J. Wei. Mesa-type patterns in the one-dimensional Brusselator and their stability. *Physica D: Nonlinear Phenomena*, 214(1):63–77, 2006. ISSN 01672789. doi: 10.1016/j.physd.2005.12.005.

15. F. Brauns, H. Weyer, J. Halatek, J. Yoon, and E. Frey. Wavelength Selection by Interrupted Coarsening in Reaction-Diffusion Systems. *Physical Review Letters*, 126(10):104101, 2021. ISSN 0031-9007, 1079-7114. doi: 10.1103/PhysRevLett.126.104101.

16. Y. Caspi and C. Dekker. Mapping out Min protein patterns in fully confined fluidic chambers. *eLife*, 5:e19271, 2016. ISSN 2050-084X. doi: 10.7554/eLife.19271.

17. A. G. Vecchiarelli, M. Li, M. Mizuuchi, and K. Mizuuchi. Differential affinities of MinD and MinE to anionic phospholipid influence Min patterning dynamics *in vitro*: Flow and lipid composition effects on Min patterning. *Molecular Microbiology*, 93(3):453–463, 2014.

ISSN 0950382X. doi: 10.1111/mmi.12669.

18. M. Loose, E. Fischer-Friedrich, C. Herold, K. Kruse, and P. Schwille. Min protein patterns emerge from rapid rebinding and membrane interaction of MinE. *Nature Structural & Molecular Biology*, 18(5):577–583, 2011. ISSN 1545-9993, 1545-9985. doi: 10.1038/nsmb.2037.

19. J. Halatek, F. Brauns, and E. Frey. Self-organization principles of intracellular pattern formation. *Philosophical Transactions of the Royal Society B: Biological Sciences*, 373 (1747):20170107, 2018. doi: 10.1098/rstb.2017.0107.
