# Supplementary material for: Bulk-surface coupling identifies the mechanistic connection between Min-protein patterns in vivo and in vitro
Source: Nat Commun. 2021 Jun 3;12:3312. doi: 10.1038/s41467-021-23412-5 (PMC8175580; doi:10.1038/s41467-021-23412-5)
Supplement: Supplementary file 23 — Reporting Summary [file 41467_2021_23412_MOESM23_ESM.pdf]

nature research

Corresponding author(s): Erwin Frey, Cees Dekker

Last updated by author(s): Feb 12, 2021

Reporting Summary

Nature Research wishes to improve the reproducibility of the work that we publish. This form provides structure for consistency and transparency in reporting. For further information on Nature Research policies, see our Editorial Policies and the Editorial Policy Checklist.

Statistics

For all statistical analyses, confirm that the following items are present in the figure legend, table legend, main text, or Methods section.

n/a	Confirmed	
x		The exact sample size (n) for each experimental group/condition, given as a discrete number and unit of measurement
x		A statement on whether measurements were taken from distinct samples or whether the same sample was measured repeatedly
x		The statistical test(s) used AND whether they are one- or two-sided<br>*Only common tests should be described solely by name; describe more complex techniques in the Methods section.*
x		A description of all covariates tested
x		A description of any assumptions or corrections, such as tests of normality and adjustment for multiple comparisons
	x	A full description of the statistical parameters including central tendency (e.g. means) or other basic estimates (e.g. regression coefficient) AND variation (e.g. standard deviation) or associated estimates of uncertainty (e.g. confidence intervals)
x		For null hypothesis testing, the test statistic (e.g. F, t, r) with confidence intervals, effect sizes, degrees of freedom and P value noted<br>*Give P values as exact values whenever suitable.*
x		For Bayesian analysis, information on the choice of priors and Markov chain Monte Carlo settings
x		For hierarchical and complex designs, identification of the appropriate level for tests and full reporting of outcomes
x		Estimates of effect sizes (e.g. Cohen's d, Pearson's r), indicating how they were calculated

Our web collection on statistics for biologists contains articles on many of the points above.

Software and code

Policy information about availability of computer code

Data collection	For acquisition of movies, tiff stacks were saved using commercial Andor software (Andor iQ3 v3.1) on an Olympus microscope IX-81 as described in the Methods section.
Data analysis	Analysis of experimental MinDE patterns was performed in ImageJ (v1.52j) Matlab (2016) using custom-written software. Details of the analysis are described in the SI. Fig. 4E,G: A003_Reslice_and_CondenseStacks.m, 510_Top_and_Bottom_Correlation_XT.m; Figs. S10,S11: A001_CleanSavePatterns, A002_ResliceStacks, A400_Correlation<br><br>The code is described in detail and publicly available on GitHub: https://github.com/jacobkers/MinED_patterns, doi:10.5281/zenodo.4637889<br><br>Numerical simulations were performed in COMSOL Multiphysics v5.4. Linear stability analysis and analysis of the numerical simulations was implemented in Mathematica. The COMSOL setup files and Mathematica notebooks are available on GitHub: https://github.com/f-brauns/Min-bulk-surface-coupling, doi:10.5281/zenodo.4644636

For manuscripts utilizing custom algorithms or software that are central to the research but not yet described in published literature, software must be made available to editors and reviewers. We strongly encourage code deposition in a community repository (e.g. GitHub). See the Nature Research guidelines for submitting code & software for further information.

Data

Policy information about availability of data

All manuscripts must include a data availability statement. This statement should provide the following information, where applicable:

- Accession codes, unique identifiers, or web links for publicly available datasets
- A list of figures that have associated raw data
- A description of any restrictions on data availability

Figures with associated data provided in the Source Data file: Figs.~3E,G and 4 and Supplementary Figs.~10, 11 and 12.
The raw data, fluorescence microscopy image sequences and exported simulation data, are available from the corresponding author on reasonable request.

Field-specific reporting

Please select the one below that is the best fit for your research. If you are not sure, read the appropriate sections before making your selection.

- [x] Life sciences
- [ ] Behavioural & social sciences
- [ ] Ecological, evolutionary & environmental sciences

For a reference copy of the document with all sections, see nature.com/documents/nr-reporting-summary-flat.pdf

Life sciences study design

All studies must disclose on these points even when the disclosure is negative.

Sample size	Fluorescence movies were analyzed for at least 5 frames per movie. For kymographs, at least 10 x- and y- cross-sections per movie were taken.
Data exclusions	No data were excluded from the analysis
Replication	For each set of conditions (MinE/MinD ratio, microchamber height) the experiments repeated 1-4 times
Randomization	All proteins used in this study are completely defined by their DNA sequence and are therefore not relevant for a randomisation process.
Blinding	The identity of all constructs and the process of analysis were clearly defined and therefore not relevant for a blinding process.

Reporting for specific materials, systems and methods

We require information from authors about some types of materials, experimental systems and methods used in many studies. Here, indicate whether each material, system or method listed is relevant to your study. If you are not sure if a list item applies to your research, read the appropriate section before selecting a response.

Materials & experimental systems

n/a	Involved in the study	
x		Antibodies
x		Eukaryotic cell lines
x		Palaeontology and archaeology
x		Animals and other organisms
x		Human research participants
x		Clinical data
x		Dual use research of concern

Methods

n/a	Involved in the study	
x		ChIP-seq
x		Flow cytometry
x		MRI-based neuroimaging
